# The Human Antiviral Factor TRIM11 Is under the Regulation of HIV-1 Vpr

**DOI:** 10.1371/journal.pone.0104269

**Published:** 2014-08-08

**Authors:** Ting Yuan, Weitong Yao, Fang Huang, Binlian Sun, Rongge Yang

**Affiliations:** Research Group of HIV Molecular Epidemiology and Virology, Center for Emerging Infectious Diseases, The State Key Laboratory of Virology, Wuhan Institute of Virology, Chinese Academy of Sciences, Wuhan, Hubei, The People's Republic of China; Institut Pasteur of Shanghai, Chinese Academy of Sciences, China

## Abstract

TRIM11 has been reported to be able to restrict HIV-1 replication, but the detailed aspects of the interfering mechanisms remain unclear. In this study, we demonstrated that TRIM11 mainly suppressed the early steps of HIV-1 transduction, resulting in decreased reverse transcripts. Additionally, we found that TRIM11 could inhibit HIV-1 long terminal repeat (LTR) activity, which may be related to its inhibitory effects on NF-κB. Deletion mutant experiments showed that the RING domain of TRIM11 was indispensable in inhibiting the early steps of HIV-1 transduction but was dispensable in decreasing NF-κB and LTR activities. Moreover, we found that low levels of Vpr decreased TRIM11 protein levels, while high levels increased them, and these regulations were independent of the VprBP-associated proteasome machinery. These results suggest that the antiviral factor TRIM11 is indirectly regulated by HIV-1 Vpr through unknown mechanisms and that the concentration of Vpr is essential to these processes. Thus, our work confirms TRIM11 as a host cellular factor that interferes with the early steps of HIV-1 replication and provides a connection between viral protein and host antiviral factors.

## Introduction

Several decades have passed since the identification of the human immunodeficiency virus (HIV) as the causative agent of acquired immune deficiency syndrome (AIDS). However, we are still unable to eradicate the virus from infected patients. Because of the difficulties that are encountered in developing traditional vaccines, investigating the potentials of antiviral factors as prophylactics or therapies will be very valuable. The replication of HIV-1 in infected cells encompasses fusion, viral core release and uncoating, reverse transcription, the translocation of the preintegration complex to the nucleus, viral DNA integration, proviral transcription and translation and viral assembly and budding [1]. Most of these steps were reported to be widely challenged by host antiviral factors, especially TRIM family members [2], [3], which share an N-terminal RING domain, one or two B-boxes, a putative coiled-coil domain and a variable C-terminus [4]. For example, the well-known restriction factor TRIM5α limits retroviral replication at multiple-steps in a species-specific manner [5], [6]. TRIM37 reduces HIV-1 DNA synthesis by being incorporated into virus particles [7], whereas TRIM28 functions via a different mechanism by inducing the deacetylation of integrase, resulting in the reduction of HIV-1 DNA integration [8]. A screening of 36 human TRIM proteins for potential anti-HIV-1 activity identified several TRIM proteins that could affect both the early and/or late stages of the virus life cycle [9]. Among those proteins, TRIM11 inhibited both the entry and release of HIV-1 [9]. However, the precise mechanisms of underlying these inhibitory functions have not yet been analyzed.

HIV-1, which is a member of the *Retroviridae* family, is characterized by the possession of viral RNA that is reverse transcribed into double stranded DNA and integrated into cellular chromosomes, generating the provirus. The transcription of the HIV-1 proviral gene is tightly regulated by *cis*-acting DNA sequences that are located within LTR region of the viral genome [10], [11]. These *cis*-acting elements provide binding sites for various cellular transcription factors, such as Sp1, NF-κB, NFAT and AP-1 [12], the availability of which contributes to the transcriptional activation of the integrated HIV-1 proviruses [7]. Thus, the HIV-1 LTR is a potential target for both the inhibition of viral replication and activation of the latent provirus. Nuclear-localized TRIM22 has been shown to impair HIV-1 replication by interfering with LTR-driven transcription in a TAT- and NF-κB-independent manner [13]. In contrast, due to the NF-κB binding sites that are located in the U3 region of the LTR, HIV-1 gene expression has been shown to be increased by TRIM proteins, such as TRIM62, that induce NF-κB; this allows for HIV-1 to replicate more efficiently than MLV despite a strong innate immune response [14]. These results raise the possibility that TRIM proteins which can negatively regulate innate immunity through NF-κB to inhibit LTR activity.

Vpr is a multifunctional accessory protein of HIV-1 that is important for the pathogenesis of HIV-1, including its ability to induce arrest at the G2-phase of cell division, induce apoptosis and modulate cell signaling [15], [16]. Vpr is also a necessary factor for HIV-1 infection in terminally differentiated macrophages [17], [18]. It is readily incorporated into virus particles through the direct interaction with the C-terminus of Gag, which indicates its enrollment during the early steps of viral replication [19]. In addition, Vpr is reported to be a component of the reverse transcription complex (RTC) and co-localizes with the viral nucleic acid and integrase within purified HIV-1 RTCs [20], [21]. Human uracil DNA glycosylase 2 (UNG2), which is an enzyme that is part of the DNA repair machinery [22], is the only protein that has been identified to date that may be involved along with Vpr in influencing HIV-1 reverse transcription [23]. However, the function of UNG2 remains controversial because various studies have reported that its impact on HIV-1 reverse transcription is negative, positive or even nil [24]–[26]. Although plenty of cellular targets of Vpr have been identified, such as UNG2, SMUG1 and ZIP [27]–[29], the importance of such targets to the restriction on HIV-1 propagation require better documentation. Therefore, unlike other HIV-1 auxiliary proteins and HIV-2 Vpx, which could rescue viral replication in certain cell types through the degradation of respective restriction factors [30]–[32], the mechanisms by which Vpr contribute to viral replication necessitate further analyses.

In the current study, we characterized the molecular roles of TRIM11 in interfering with HIV-1 transduction. In addition, we found that the HIV-1 Vpr protein indirectly regulates TRIM11 protein levels by means that are unrelated to the proteasomal system. In these processes, the concentration of Vpr was important for the direction of regulation. Thus, our study provides the possibility that Vpr may utilize new mechanisms to control cellular antiviral factors.

## Materials and Methods

### Cell culture, reagents and antibodies

Human embryonic kidney cells (HEK293 and HEK293T) were maintained in Dulbecco's modified Eagle's medium (Gibco, Auckland, NZ) containing 10% fetal calf serum (Gibco).

The antibodies and reagents that were used in this work and their sources were as follows: anti-VprBP (Santa Cruz Biotechnology, Dallas, TX), anti-actin, anti-TRIM11, anti-Myc, anti-Flag and anti-HA (Sigma-Aldrich, St. Louis, MO) and puromycin and MG132 (Sigma-Aldrich).

### Plasmid constructs

The cDNA for TRIM11 was obtained by PCR using total cDNA that was prepared from HEK293 cells as a template. The PCR product was cloned into a pCMV-Myc vector to construct the Myc-TRIM11 (Myc-T11) expression vector. Using the cloned Myc-T11 as a template, mutant TRIM11 lacking the RING domain was amplified by PCR and cloned into the pCMV-Myc vector to construct a RING-domain-deleted TRIM11 expression vector. The NF-κB and LTR promoter luciferase reporter plasmids were generated as previously described [33]. The Flag-Vpr expression vector was kindly provided by Dr. Y. Ishizaka (National Center for Global Health and Medicine, Japan).

### Generation of stable cell lines

A C-terminal HA-tagged TRIM11 and HA-tagged RING domain deletion TRIM11 were obtained by PCR using Myc-T11 as a template, and the PCR products were cloned into the lentiviral expression vector, pCDH-CMV-MCS-EF1-Puro (System Biosciences, North Shorelin Blvd, CA). Recombinant viruses were produced in the HEK293T cells by co-transfecting pCDH-HA-TRIM11 (HAT11), pCDH-HA-RDTRIM11 (HARDT) or empty pCDH with the pVSV-G and p8.9 packaging plasmids. Culture supernatants were harvested and filtered (0.45-µm pore size) at 48 h post-transfection and used for the infection of the HEK293 cells. Twenty-four hours after infection, the HEK293 cells were selected in medium containing 1.3 µg/ml puromycin and maintained in 0.85 µg/ml puromycin.

A lentiviral vector pKLO.1 system (Sigma-Aldrich) was used to stably knock down target gene expression. pLKO.1-shRNA plasmids expressing TRIM11 shRNA, VprBP shRNA and GFP shRNA (negative control) were constructed with targeting sequences as follows: TRIM11 CCGGGAGCTGATCCTGTCTGAAGTTCTCGAGAACTTCAGACAGGATCAGCTCTTTTTG, VprBP CCGGCGAGAAACTGAGTCAAATGAACTCGAGTTCATTTGACTCAGTTTCTCGTTTTTG, and GFP CCGGTTCATCTGCACCACCGGCAAGCCTCGAGGCTTGCCGGTGGTGCAGATGATTTTTG. The HEK293T cells were transfected with each pLKO.1-shRNA construct along with the packaging plasmids psPAX2 and pMD.2G. The shRNA lentiviral stocks were harvested from the culture medium at 48 h post-transfection and filtered using a 0.45 µm filter. The selection of HEK293 cells that stably expressed the shRNA constructs were conducted as described above.

### Real-time PCR

Total viral DNA, 2-LTR circle DNA and integrated viral DNA were analyzed as previously described [34]. To avoid any cross reaction with the viral DNA, the total and integrated HIV-1 DNA analyses of the cells carrying the pCDH and pLKO.1-shRNA lentiviral expression vectors were performed using primers that were specific for the luciferase gene at 24 h or 15 days post-infection, respectively [35]. Primers that were specific for the luciferase gene and 2-LTR circle DNA are as follows: *Luciferase*-GGCGCGTTATTTATCGGAGTT (forward) and CAACCCCTTTTTGGAAACAAAC (reverse); and 2-LTR- GTGCCCGTCTGTTGTGTGACT (forward) and ACTGGTACTAGCTTGTAGCACCATCCA (reverse).

### Dual luciferase assay

The HEK293 cells were seeded in 12-well plates and transfected the following day with the corresponding plasmids using X-treme HP (Roche, Indianapolis, IN). In addition, 50 ng of thymidine kinase (TK)-*Renilla* luciferase reporter plasmids (pRL-TK) were mixed into each reaction for the normalization of the transfection efficiencies. Twenty-four hours after transfection, dual-specific luciferase reporter gene assays were performed using the Dual-Luciferase Reporter Assay System in accordance with the manufacturer's instructions (Promega, Madison, WI). Firefly luciferase activity was normalized based on the activity of *Renilla* luciferase.

### Single-cycle infectivity assay

The HIV-1 pNL4-3.Luc.R+E- or pNL4-3.Luc.R-E- plasmids, which were provided by Dr. K. Tokunaga (National Institute of Infectious Diseases, Japan), were cotransfected with pVSVG using X-treme HP (Roche) to produce Vpr-positive or Vpr-negative pseudotyped HIV-1 NL4-3.Luc viruses (HIV-1 Vpr^+^ and HIV-1 Vpr^−^). Virus supernatants were harvested and filtered (0.45-µm pore size) at 48 h post-transfection. The viral titers were measured with a p24 ELISA kit (Advanced BioScience Laboratories, Inc). Cell lines carrying the pCDH and pLKO.1-shRNA lentiviral expression vectors were seeded in 48-well plates and infected with different amounts of HIV-1 Vpr^−^. The cells were collected at 24 h post-infection for the measurement of luciferase activity (Promega), which was normalized by the protein OD of each sample.

### siRNA knockdown

All siRNAs targeting *TRIM11* and the control siRNA (Si-Ctrl) were purchased from RiboBio (GuangZhou RiBo Biotech Co., Ltd.). HEK293 cells were seeded in 12-well plates the day before transfection. For the single-cycle infectivity assay, cells that were transfected with 50 nM *TRIM11*-specific siRNA or control siRNA using Lipofectamine 2000 (Invitrogen) were infected with HIV-1 Vpr^−^ as described above at 24 h after transfection. After an additional 24 h culture, the cells were harvested and analyzed for virus infectivity. For the dual-luciferase activity assays, cells were transfected with *TRIM11*-specific siRNA or control siRNA together with 50 ng of the LTR luciferase reporter construct and 50 ng of pRL-TK using Lipofectamine 2000. After 24 h, the cells were harvested and analyzed by the dual-luciferase activity assay as described above. The medium of the cells transfected with siRNA was changed at 4 h post-transfection.

### Co-immunoprecipitation (Co-IP) assay

The HEK293 cells were transfected in 100-mm dishes with the indicated plasmids. Twenty-four hours after transfection, the cells were collected and washed twice with 1×PBS, resuspended gently in 450 µl IP buffer and placed on a vibrating mixer at 4°C for 20 min. The cell lysates were then centrifuged, and the supernatants were separated into two aliquots. One aliquot of 400 µl was mixed with the pre-washed protein G agarose (Merck Millipore, Darmstadt, Germany) together with 1 µg of the indicated antibodies (control mouse IgG and anti-Myc or anti-HA MAb) and placed on a rotational tumbler for incubation overnight at 4°C. The agarose was washed with IP buffer and boiled with 50 µl Laemmli sample buffer and designated as the IP sample. Another aliquot of 40 µl supernatant was boiled with sample loading buffer (5×) (Beyotime, Shanghai, China) and designated as the input sample. All of the samples were analyzed by immunoblotting.

### Cell viability

HEK293 cells were cultured in 96-well plates with 100 µl medium per well and transfected with 900 ng Myc-TRIM11 or pCMV-Myc for a certain period of time. MTT assay was conducted in accordance with the manufacturer's instructions (Beyotime).

### Statistical Analyses

The data are presented as the means ± SD of three independent experiments that were performed in triplicate, and all data were analyzed using Student's t-test. P<0.05 (*) was considered to be statistically significant.

## Results

### TRIM11 inhibits HIV-1 transduction by interfering with the early steps of viral transduction

**Figure 1:**
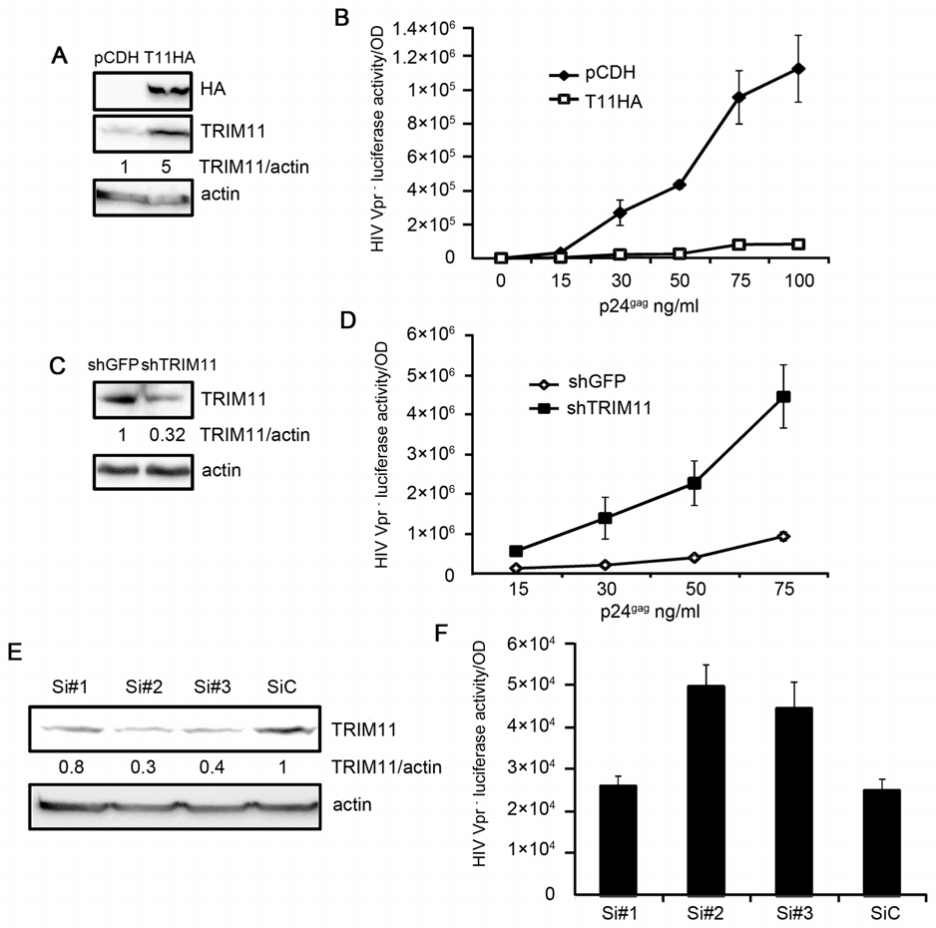
Effects of TRIM11 on HIV-1 transduction. **A**. Lysates from the HEK293 cells that were stably transduced with either pCDH or pCDH-TRIM11 were subjected to a western blot with the indicated antibodies. The numbers under each line display the relative ratios between the TRIM11 signals and actin signals. **B**. HEK293 cells stably expressing TRIM11 or a control pCDH vector were inoculated with various amounts of HIV-1 Vpr^−^ viruses. Luciferase assays were performed at 24 h post-infection (hpi). **C**. Lysates from HEK293 cells that were stably transduced with shRNA targeting TRIM11 or GFP were subjected to a western blot with the indicated antibodies. The numbers under each line display the relative ratios between the TRIM11 signals and actin signals. **D**. TRIM11 knock-down and control cell lines were inoculated with various amounts of HIV-1 Vpr^−^ viruses. Luciferase assays were performed at 24 hpi. **E**. Western blot analysis of TRIM11 expression in HEK293 cells transfected with control siRNA or TRIM11 siRNA#1, siRNA#2 and siRNA#3 for 24 h. The number under each line displays the relative ratios between the TRIM11 signals and actin signals. **F**. HEK293 cells transfected with control siRNA or TRIM11 siRNA were inoculation with 50 ng/ml p24^gag^ HIV-1 Vpr^−^ virus. Luciferase activity assays were performed at 24 hpi. Error bars represent the standard deviations from three independent replicates of the same experiment.

TRIM11 has been identified to potently interfere with HIV-1 replication in a screening of TRIM proteins that were transiently expressed in HEK293 cells [9]. To confirm these results, we constructed two HEK293 cell lines that stably expressed HA-TRIM11 or a control vector pCDH (Figure 1A). We first performed cell proliferation assay by counting cell numbers and found that cells stably expressing TRIM11 had minimal effect on cell proliferation (Figure S1A). The two cell lines were then inoculated with different amounts of HIV-1 Vpr^−^ viruses, and the luciferase activities of the infected cells w examined to confirm the infection levels. In contrast with the control vector, the overexpression of TRIM11 resulted in a marked inhibition of HIV-1 transduction (Figure 1B). To assess whether TRIM11 expression at physiological levels possesses antiviral activity, we also constructed a knockdown cell line that stably expressing short hairpin ribonucleic acid (shRNA) that is directed against TRIM11. Because GFP is not expressed in mammalian cells, the cell line stably expressing shRNA that targets GFP served as the non-targeting control. As shown in Figure 1C, compared with the control cells, TRIM11 protein levels were significantly reduced in the knockdown cell line. Luciferase activity was monitored at 24 h after the inoculation of different amounts of HIV-1 Vpr^−^ viruses in these two cell lines. The results showed that the TRIM11 knockdown cell line facilitated HIV-1 transduction by an average of approximately seven-fold (Figure 1D). To confirm the effect of endogenous TRIM11 on HIV-1 transduction, we knocked down TRIM11 expression by siRNAs. Three specific siRNAs for TRIM11 and a control siRNA were transfected into HEK293 cells. Western blot analysis showed that only siRNA#2 and siRNA#3 have apparent effects on reducing TRIM11 compared with the control siRNA. Single-round infectivity assay revealed that knockdown of TRIM11 by specific siRNA also facilitated HIV-1 transduction (Figure 1F). Taken together these results suggest that TRIM11 exhibits an inhibitory activity on HIV-1 transduction.

**Figure 2:**
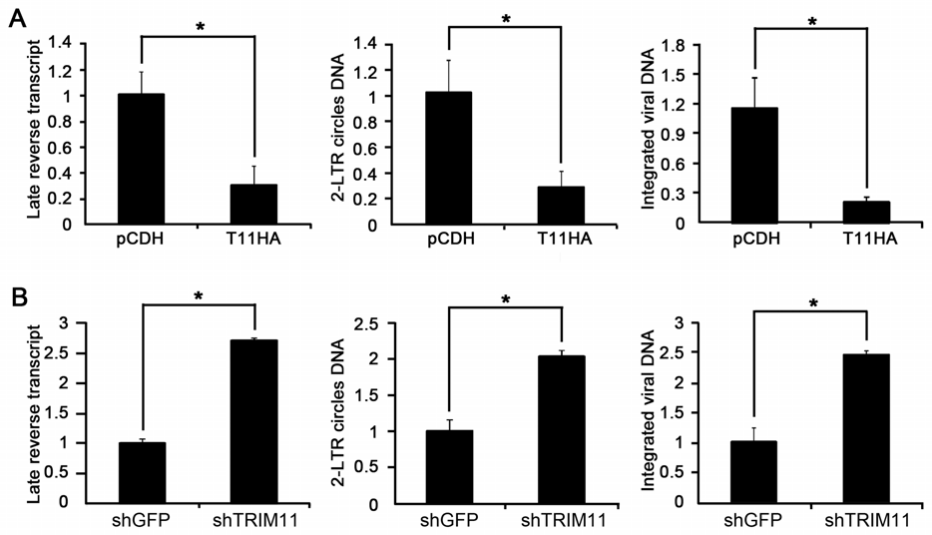
Effects of TRIM11 on the early steps of HIV-1 replication. **A**. HEK293 cells stably expressing TRIM11 or control pCDH vector were inoculated with 50 ng/ml (p24^gag^) of HIV-1 Vpr^−^ viruses and analyzed by qPCR for late reverse transcripts and 2-LTR circle DNA at 24 hpi and integrated DNA at 14 day post infection (dpi). **B**. TRIM11 knock-down and control cell lines were inoculated with 50 ng/ml (p24^gag^) of HIV-1 Vpr^−^ viruses and analyzed by qPCR for late reverse transcripts and 2-LTR circle DNA at 24 hpi and integrated DNA at 14 dpi. Error bars represent the standard deviations from three independent replicates of the same experiment. *P<0.05.

The transduction of HIV-1 in this study encompassed several events of the retroviral life cycle. To examine which events TRIM11 interfered with, we infected cell lines that stably expressed control vector or HA-TRIM11 with 50 ng/ml (p24^gag^) of HIV-1 Vpr^−^, and the different forms of the viral DNA (late reverse transcripts, 2-LTR DNA and integrated DNA) in the infected cells were quantified with relatively quantitative PCR. Surprisingly, the levels of all three forms of viral DNA were significantly lower in the TRIM11 overexpression cell line compared with the control cell line (Figure 2A). These results indicate that ectopic TRIM11 expression most likely inhibits the events of the HIV-1 replication cycle before retrotranscription, resulting in decreased late reverse transcripts and diminishing the levels of 2-LTR circle DNA and integrated viral DNA. Furthermore, we confirmed the above results using a TRIM11 knockdown cell line. Reducing TRIM11 can enhance the levels of viral reverse transcripts, 2-LTR circle DNA and integrated viral DNA (Figure 2B). In conclusion, TRIM11 may interfere with HIV-1 transduction by restricting the early steps before retrotranscription.

### TRIM11 can inhibit HIV-1 LTR activity in a manner that is partially dependent on the NF-κB pathway

**Figure 3:**
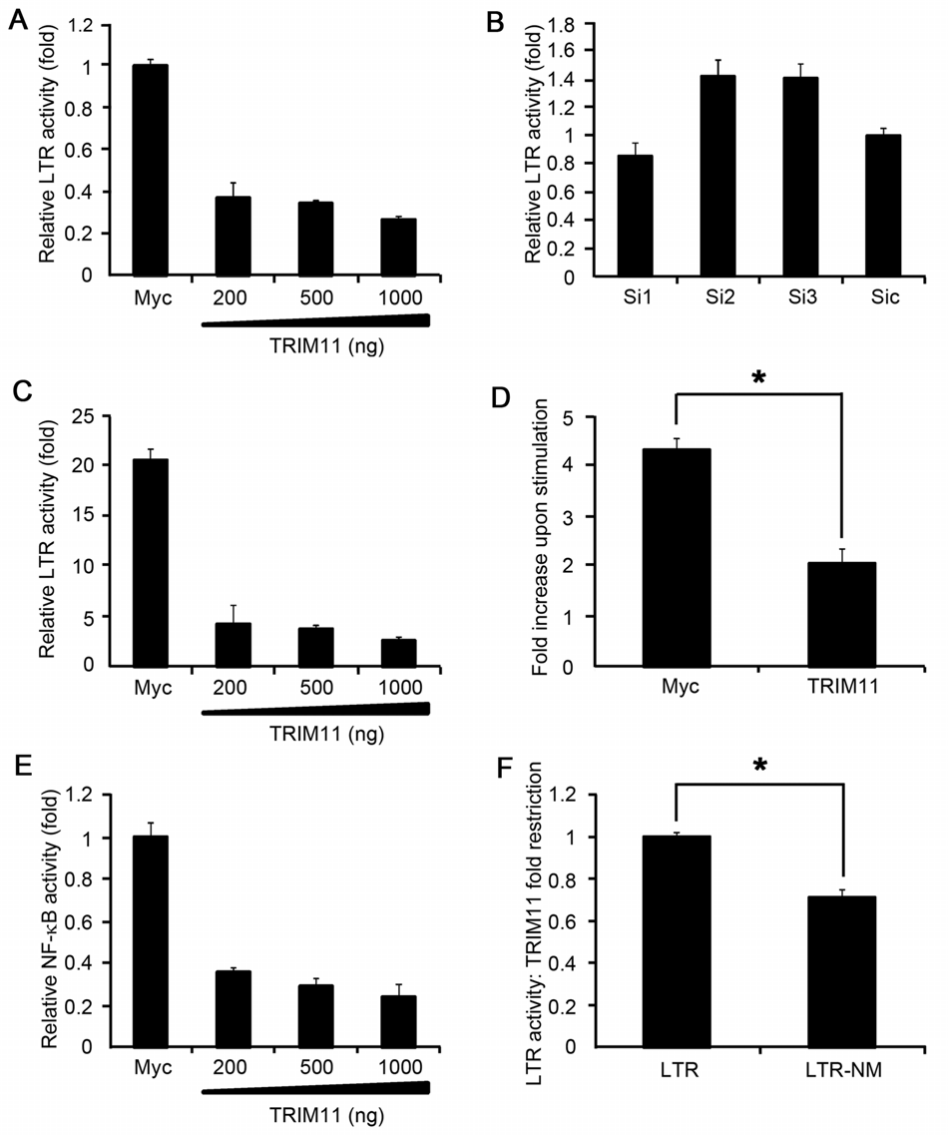
Effects of TRIM11 on HIV-1 LTR activity. **A, C**. HEK293 cells were cotransfected with 200, 500 or 900 ng of plasmids expressing TRIM11, 50 ng of the HIV-1 LTR firefly luciferase reporter and 50 ng of *renilla* luciferase plasmid. Twenty hours after transfection, cells were left untreated (A) or were treated with 20 ng/ml TNFα(C) for 4 h before luciferase assays were performed. **B**. HEK293 cells were cotransfected with control siRNA or TRIM11 siRNA, 50 ng of the HIV-1 LTR firefly luciferase reporter and 50 ng of *renilla* luciferase plasmid. Luciferase assays were performed at 24 h after transfection. **D**. Fold inductions upon TNFα stimulation were compared in the absence (Myc) or presence of 900 ng of Myc-TRIM11. **E**. HEK293 cells were cotransfected with 200, 500 or 900 ng of plasmids expressing TRIM11, 50 ng of the NF-κB firefly luciferase reporter and 50 ng of *renilla* luciferase plasmid. Luciferase assays were performed at 24 h after transfection. **F**. The wild type or NF-κB mutant LTR reporter constructs along with the 900 ng Myc-TRIM11 plasmids or empty vectors were transfected into HEK293 cells. Luciferase assays were performed at 24 h after transfection. Data are presented as fold-changes compared with TRIM11 restriction rates on the wild type LTR promoter. Error bars represent the standard deviations from three independent replicates of the same experiment. *P<0.05.

HIV-1 transduction, which was measured in terms of luciferase activity as shown in Figure 1, encompassed not only the early steps but also the LTR-directed transcription. We further investigated whether TRIM11 affected HIV-1 transcription under the LTR promoter by cotransfecting HEK293 cells with increasing amounts of the TRIM11-expressing vector together with a fixed amount of the HIV-1 LTR luciferase reporter construct. The results from the dual luciferase assay showed that TRIM11 significantly decreased HIV-1 LTR activity as its concentration increased (Figure 3A). The effect of ectopically expressed TRIM11 on cell viability was assessed by MTT assay (Figure S1B). Compared with control vector, overexpression of TRIM11 did not show any additional effect on cell viability (Figure S1B). To determine the effects of endogenous TRIM11 on HIV-1 LTR activity, we knocked down TRIM11 by siRNAs. A dual luciferase assay revealed that the knockdown of TRIM11 correspondingly facilitated HIV-1 LTR activity by approximately 1.5-fold (Figure 3B). These results suggest that TRIM11 is able to inhibit basal HIV-1 transcription.

**Figure 4:**
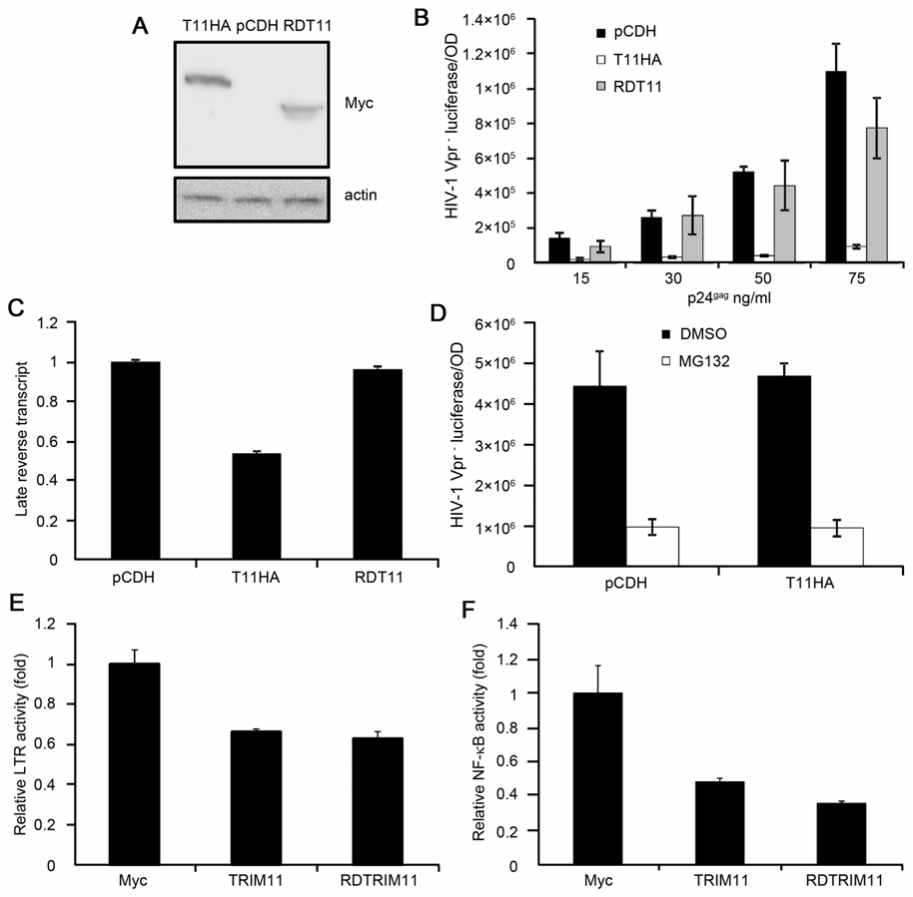
Effects of the RING domain and proteasome system on the functioning of TRIM11. Lysates from HEK293 cells stably transduced with pCDH, pCDH-TRIM11 or pCDH-RDTRIM11 were subjected to a western blot with the indicated antibodies. **B**. HEK293 cells stably expressing TRIM11, RDTRIM11 or a control pCDH vector were inoculated with various amounts of HIV-1 Vpr^−^ viruses. Luciferase assays were performed at 24 hpi. **C**. HEK293 cells stably expressing TRIM11, RDTRIM11 or control pCDH vector were inoculated with 50 ng/ml (p24^gag^) of HIV-1 Vpr^−^ viruses and were analyzed by qPCR for viral DNA. **D**. HEK293 cells stably expressing TRIM11, RDTRIM11 or control pCDH vector were pretreated with MG132 or DMSO for 5 h and inoculated with 50 ng/ml (p24^gag^) of HIV-1 Vpr^−^ viruses. Luciferase assays were performed at 14 hpi. **E, F**. HEK293 cells were cotransfected with 900 ng of plasmids expressing TRIM11, RDTRIM11 or control vector along with 50 ng of the HIV-1 LTR firefly luciferase reporter (E) or 50 ng of the NF-κB firefly luciferase reporter (F) and 50 ng of *renilla* luciferase plasmid. Luciferase assays were performed at 24 h after transfection. Error bars represent the standard deviations from three independent replicates of the same experiment.

TNFα is known be able to activate HIV-1 transcription [11], [36]. Therefore, HEK293 cells were stimulated with TNFα for an additional 4 h after transfection with the TRIM11 overexpression vector or control vector and with the HIV-1 LTR luciferase reporter construct for 24 h. Increasing amounts of TRIM11 also led to decreases in TNFα-stimulated HIV-1 LTR activity (Figure 3C). To determine whether the inhibitory effects of TRIM11 on TNFα-induced LTR activation could be due to impaired basal transcription (Figure 3A), we examined the fold increase in luciferase activity that was observed upon stimulation in both the control and TRIM11- transfected cells. In comparison with the control cells, the TRIM11-overexpressing cells had significantly lower LTR activities in terms of fold induction versus baseline (Figure 4D). These results indicate that TRIM11 inhibited both basal and TNFα-induced HIV-1 LTR transcription.

Because TRIM11 has been proven to negatively regulate RIG-I induced NF-κB activity, we assessed whether it contributed to the TRIM11 inhibition of HIV-1 LTR activity. First, we cotransfected HEK293 cells with increasing amounts of the TRIM11-expression vector together with a fixed amount of the NF-κB luciferase reporter construct. The dual luciferase assay confirmed that the overexpression of TRIM11 in HEK293 cells hindered NF-κB activity (Figure 3E). Next, we introduced an LTR with a mutation in the two NF-κB binding sites to the luciferase construct. Figure 3F shows that the mutant LTR compromised the TRIM11 restriction activity to levels that were approximately 70% of the wild type LTR. These results indicate that other than restricting the early steps of HIV-1 transduction, TRIM11 may also inhibit HIV-1 LTR activity in a manner that is partially dependent on the NF-κB pathway.

### The RING domain of TRIM11 is necessary for its antiviral activity but dispensable for its negative regulation on LTR and NF-κB activity

As an E3 ubiquitin ligase, TRIM11 has been reported to mediate the ubiquitination of several target proteins [37]–[40]. We next tested the contribution of the E3 ligase function of TRIM11 to the inhibition of HIV-1 transduction. We constructed another cell line that stably expressed a RNIG-deleted mutant of TRIM11, which was also demonstrated having little effect on cell proliferation (Figure S1A). Figure 4A shows the expression levels of the wild type and mutant TRIM11 in the two cell lines. A single-cycle infectivity assay showed that although there were differences when using high concentrations of the virus, the transduction of HIV-1 in the mutant TRIM11-overexpression cell line was relatively similar to that in the control cell line, and it was markedly higher than that in the full-length TRIM11-overexpression cell line (Figure 4B). Furthermore, when the cell lines were infected with 50 ng/ml (p24^gag^) of HIV-1 Vpr^−^ viruses, the overexpression of the mutant TRIM11 fully restored viral reverse transcripts compared with the full-length TRIM11 (Figure 4C). These results suggest that the RING domain of TRIM11 plays an indispensable role in its antiviral activity, specifically in decreasing the abundance of late reverse transcripts.

It has been reported that the RING domain of TRIM11 contributes to destabilizing target proteins through proteasomes as a functional E3 ubiquitin ligase [37], [38]. To test whether the inhibitory function of TRIM11 on viral transduction depends on the proteasome, we pretreated the cells with the proteasome inhibitor MG132 for 5 h before the addition of 50 ng/ml (p24^gag^) of HIV-1 Vpr^−^ viruses. The luciferase activity indicated that the inhibition of the proteasomes by MG132 could not restore the viral transduction (Figure 4D), suggesting that the contribution of the RING domain to the TRIM11-directed inhibition of virus transduction does not occur through the proteasome pathway.

In contrast with the reductions in viral reverse transcripts, the inhibition of HIV-1 LTR activity was fully sustained by the RING-deleted mutant TRIM11 (Figure 4E), which may contribute to the marginal reduction of luciferase activity at high levels of HIV-1 infection. Moreover, the mutant TRIM11 also showed an intact negative regulation on NF-κB activity compared with the full-length TRIM11 (Figure 4F), suggesting that its inhibition on LTR activity may be related to its ability to negatively regulate NF-κB. Collectively, these results suggest that TRIM11 could interfere with both the early steps of virus replication and HIV-1 LTR activity, which may occur through completely different mechanisms.

### Concentration-dependent regulation of TRIM11 protein levels by HIV-1 Vpr

**Figure 5:**
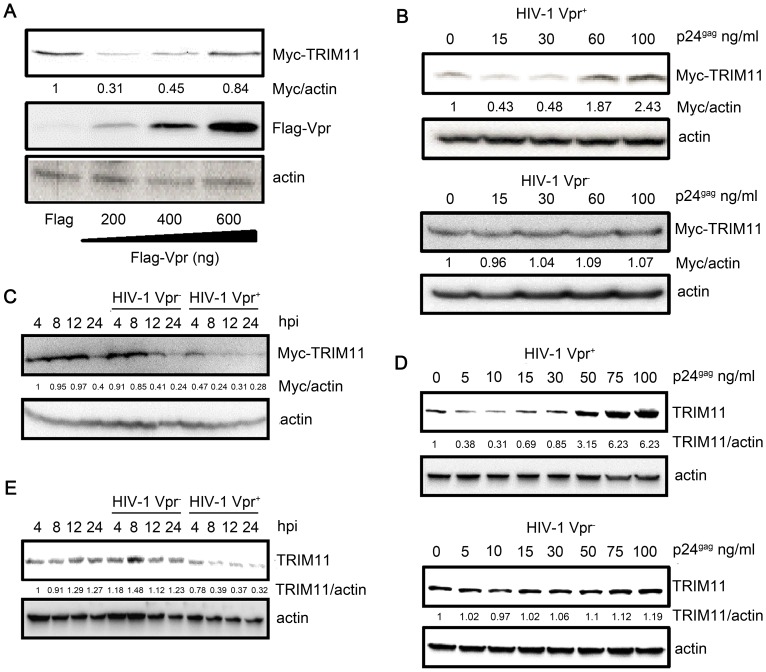
Effects of Vpr on TRIM11 protein levels. **A**. HEK293 cells were cotransfected with TRIM11 expression plasmids and increasing amounts of Vpr expressing plasmids for 24 h and cell lysates were immunoblotted with the indicated antibodies. **B, D**. HEK293 cells that were transfected with TRIM11-expressing plasmids (B) for 24 h or not (D) were inoculated with increasing amounts of HIV-1 Vpr^−^ or HIV Vpr^+^ virus. Cell lysates were immunoblotted with the indicated antibodies at 12 hpi (B) or 24 hpi (D). **C, E**. HEK293 cells that were transfected with TRIM11-expressing plasmids (C) for 24 h or not (E) were inoculated with 20 ng/ml (p24^gag^) of HIV-1 Vpr^−^ and HIV-1 Vpr^+^ viruses for different periods of time. Cell lysates were immunoblotted with the indicated antibodies. The numbers under each line display the relative ratios between the Myc or TRIM11 signals and actin signals. Representative results from three separate experiments are shown.

To determine whether any HIV-1 protein plays a role in antagonizing TRIM11 activity, we examined the effects of all HIV-1 proteins on TRIM11. The results indicated that among the six expressed proteins (Tat, Nef, Pol, Vif, Rev and Vpr), only Tat and Vpr could decrease TRIM11 protein levels (Figure S2A). To confirm these results, we cotransfected HEK293 cells with increasing amounts of Vpr or Tat and a fixed amount of the TRIM11-expression vector. Interestingly, the transfection of 200 ng of the Vpr-expression vector severely down-regulated TRIM11, while its protein levels were recovered as the concentration of Vpr increased (Figure 5A). Tat only affected TRIM11 protein levels weakly in a dose-dependent manner (Figure S2B).

We were particularly interested in the mode of action of Vpr on TRIM11. Thus, to investigate the effects of Vpr on TRIM11 during HIV-1 infection, we infected cells that were transiently overexpressing TRIM11 with the HIV-1 Vpr^−^ or Vpr^+^ virus. The results of the dose-course infection experiments were consistent with the results from the Vpr and TRIM11 cotransfection experiment (Figure 5A). Compared with Vpr^−^, Vpr^+^ virus infection down-regulated TRIM11 protein levels at lower concentrations while it up-regulated these levels at higher concentrations (Figure 5B). We also conducted an HIV-1 infection time-course experiment using a relatively low concentration (p24^gag^, 20 ng/ml) of virus. As expected, the results showed that Vpr^+^ virus infection profoundly decreased TRIM11 protein levels, beginning at 4 h post-inoculation, indicating that the effects of Vpr during HIV-1 infection may not depend on viral genome integration and transcription (Figure 5C). To determine the effects of Vpr on endogenous TRIM11 levels, we repeated the HIV-1 infection dose-course and time-course experiments in the HEK293 cells. Similar to the results involving the ectopic expression of TRIM11 (Figure 5B), endogenous TRIM11 also decreased with lower levels of Vpr^+^ virus infection but increased with higher levels of infection (Figure 5D). A time-course experiment with low levels of Vpr also showed the down-regulation of endogenous TRIM11 (Figure 5E). Collectively, these results indicate that different concentrations of Vpr in cells can regulate TRIM11 protein levels bidirectionally.

### The regulation of TRIM11 by Vpr is independent of the ubiquitin system

**Figure 6:**
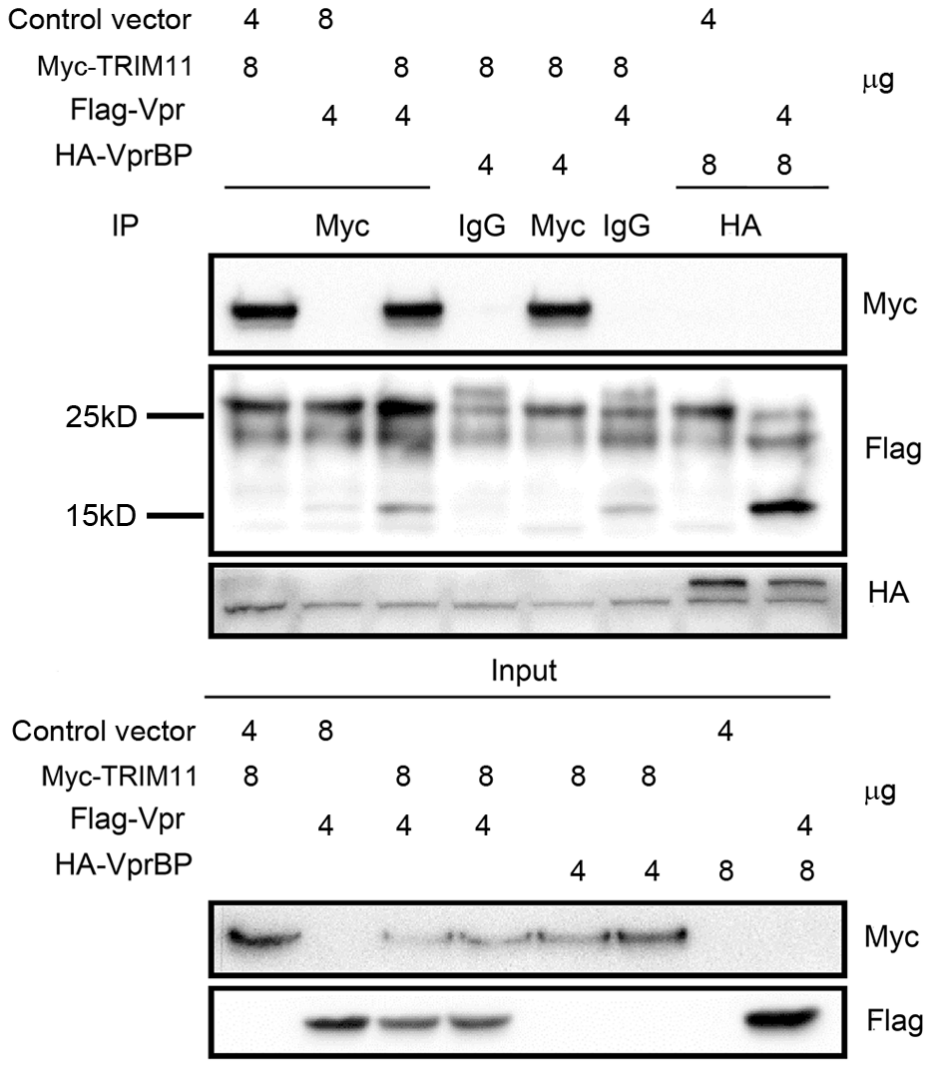
Interactions between TRIM11 and Vpr or VprBP. HEK293 cells were cotransfected with the indicated combinations of expression vectors (TRIM11-Myc, Vpr-Flag and VprBP-HA). Twenty-four hours post-infection cell lysates were immunoprecipitated and immunoblotted with the indicated antibodies. Representative results from three separate experiments are shown.

**Figure 7:**
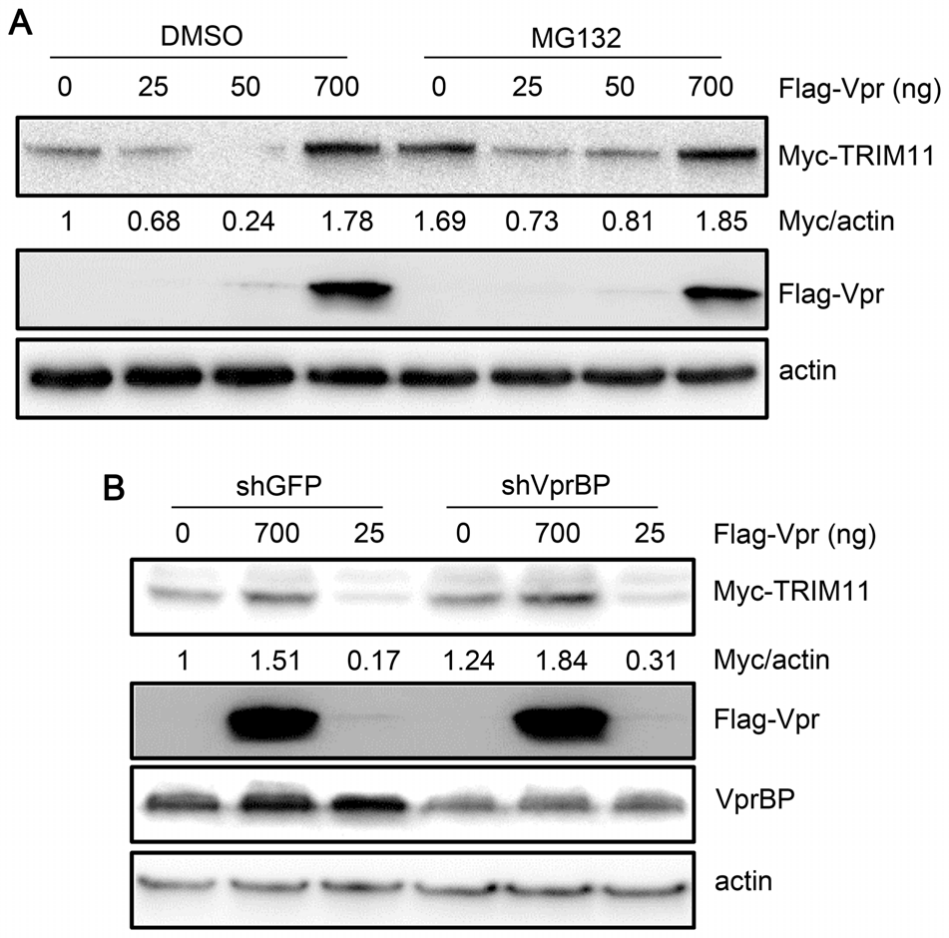
Effects of proteasomes and VprBP on the regulation of TRIM11 by Vpr. **A**. HEK293 cells were cotransfected with different amounts of Vpr and 300 ng Myc-TRIM11. Twelve hours post-transfection, cells were treated with DMSO or MG132 for another 12 h. Cell lysates were immunoblotted with the indicated antibodies. **B**. HEK293 cells stably transduced with shRNA targeting VprBP or GFP were cotransfected with different amounts of Vpr and 300 ng Myc-TRIM11. Twenty-four hours post-transfection, cell lysates were immunoblotted with the indicated antibodies. The numbers under each lines display the relative ratios between the Myc signals and actin signals. Representative results from three separate experiments are shown.

Recently, the fact that Vpr is able to bridge the proteasome to degrade a variety of substrates by binding to the E3 ubiquitin ligase complex has attracted attention [27]–[29]. Vpr binding protein (VprBP), which is also known as DDB1-Cul4A associated factor 1 (DCAF1), acts as an adaptor to link Vpr to the CUL ubiquitin E3 ligase system [41], [42]. Several proteins, including UNG2 and SMUG1, have been reported to be targets of Vpr through this proteasome system [27], [28]. We therefore tested whether Vpr or VprBP could interact with TRIM11 by co-immunoprecipitation. As shown in Figure 6, we failed to see any interaction between TRIM11 and either Vpr or VprBP. The interaction between Vpr and VprBP was used as a positive control to assess the experimental system. Thus, we deduced that Vpr may indirectly regulate TRIM11 protein levels. To determine whether this regulation was dependent on the ubiquitin system or VprBP-mediated pathway as the degradation of UNG2 and SMUG1 by Vpr, we first assessed the effects of different amounts of Vpr on TRIM11 following DMSO or MG132 treatments. In accordance with the above results, Vpr was able to down-regulate TRIM11 protein levels at lower concentrations, and up-regulate these levels at higher concentrations (Figure 7A). However, we failed to see significant differences in TRIM11 protein levels following treatment with DMSO or MG132 when Vpr was expressed at different concentrations (Figure 7A). In addition, TRIM11 protein levels profoundly increase following MG132 treatment in the absence of Vpr (Figure 7A), indicating that its protein levels may have been in a balance between degradation and synthesis, similar to other TRIM family members [43]. These results suggest that the regulation of TRIM11 by Vpr may be independent of the ubiquitin system. Furthermore, we tested the effects of VprBP on the regulation of TRIM11 by Vpr. We constructed another knockdown cell line stably expressing shRNA that targeted VprBP. Then, we cotransfected Myc-TRIM11 and different amounts of Vpr into a VprBP-knockdown cell line and control cell line expressing shRNA for GFP. As shown in Figure 7B, the knockdown of VprBP did not compromise the regulation of Vpr on TRIM11. In contrast, TRIM11 protein levels mildly increased in the VprBP-knockdown cell line and were irrelevant to Vpr expression (Figure 7B). Together, these results suggest that Vpr may indirectly regulate TRIM11 in a concentration-related manner, which is independent of the ubiquitin system or VprBP-mediated pathway.

## Discussion

A study of the screening for potential antiviral activities of human and mouse TRIM proteins found that TRIM11 could modulate both the entry and release of HIV-1 replication [9]. TRIM11 expression levels in activated PBMCs have been shown to be correlated with impaired virus replication using an *in vitro* standardized HIV-1 replication assay [44], which indicates that TRIM11 may act as a cell-intrinsic anti-HIV-1 factor during viral propagation. However, the molecular mechanism underlying the inhibitory effects of TRIM11 on HIV-1 replication is unclear. Our study showed that TRIM11 can decrease late reverse transcripts of HIV-1 and repress HIV-1 LTR activity to some extent. More importantly, our results imply that the HIV-1 accessary protein, Vpr, can regulate TRIM11 protein levels during infection.

The RING-deleted mutant TRIM11 showed the full restoration of HIV-1 reverse transcripts at 50 ng/ml (p24^gag^) of HIV-1 infection (Figure 4C), suggesting that the RING-domain of TRIM11 is necessary for restricting the early steps of HIV-1 transduction. Additionally, this mutant TRIM11 was nearly able to restore HIV-1 infectivity to the level of the control cells (Figure 4B), whereas it continued to inhibit HIV-1 LTR activity relative to the full-length TRIM11. We observed a clear difference in HIV-1 infectivity between the control and RING-deleted mutant TRIM11 expression cell lines at 75 ng/ml (p24^gag^) of HIV-1 infection, which may have resulted from the retaining of the inhibition of the mutant TRIM11 on LTR activity. However, we cannot rule out the possibility that the inhibition of reverse transcripts was partially sustained by the mutant TRIM11 at this concentration of viral infection. In fact, antiviral factors may restrict HIV-1 replication by different mechanisms under varying circumstances. TRIM22 blocked the release of HIV-1 particles in HOS-CD4/CXCR4 cells, which was dependent on the RING domain [45]. In contrast, Kajaste-Rudnitski et al. found that TRIM22 restricted HIV-1 replication by inhibiting LTR-driven transcription in a RING domain-independent manner in U937 cells [13]. Whether the inhibition of TRIM11 on LTR activity would contribute to the restriction of HIV-1 transduction in other cell types requires further investigation.

Another TRIM family member that could restrict retroviral replication before reverse transcription, TRIM5α, was demonstrated specifically to recognize the viral capsid and promote the uncoating of the virus, ultimately resulting in decreased reverse transcripts [46]. This process required the E3 ubiquitin ligase activity of the RING domain of TRIM5α to facilitate the higher-order association of its dimers for capsid binding [47]. Our results also demonstrated that TRIM11 could lead to decreased HIV-1 reverse transcripts and that the RING domain was necessary for the inhibitory activity of TRIM11 during this step. As an E3 ubiquitin ligase, TRIM11 has been found to destabilize different targets in separate biological processes through the ubiquitin-proteasome system [37]–[40]. In the case of its inhibition of HIV-1 transduction, the function of the RING domain of TRIM11 is not dependent on the ubiquitin proteasome system, demonstrated by the MG132 treatment in our study (Figure 4D). Additionally, these results imply that the exact contribution of TRIM11 to the decrease viral reverse transcripts is related to a separate unknown function of the RING domain.

In the interaction between HIV-1 and human cells, HIV-1 must take advantage of multiple host proteins, such as cyclinT1 and CRM1, to successfully replicate because the virus only encodes a limited number of proteins [48]. These viral proteins have evolved to neutralize the effects of host antiviral factors or to evade the host immune response [30]–[32]. In this study, we found that low levels of transfected or virus containing Vpr decreased, whereas high levels increased, both the exogenous and endogenous TRIM11 protein levels. We also demonstrated that TRIM11 acted as an antiviral factor during HIV-1 transduction. Therefore, our results suggest the possibility that Vpr may influence the early stages of HIV-1 transduction by regulating TRIM11. However, Vpr only slightly increased HIV-1 replication in HEK293 cells regardless of its concentrations (Figure S3A). A previous study found that Vpr plays a more important role in enhancing the nuclear import of HIV-1 DNA than the reverse transcription of a single-cycle HIV-1 infection in dividing cells [49]. Furthermore, the inhibition rates of TRIM11 on Vpr^−^ and Vpr^+^ virus infection were nearly equal (Figure S3B). The failure of Vpr to regulate HIV-1 replication by affecting TRIM11 protein levels may have been because in this single-cycle infectivity assay, the inhibition by TRIM11 of early events could possibly take place before regulation by Vpr. Therefore, TRIM11 may be down-regulated by Vpr after decreasing reverse transcripts. In contrast with its minimal contribution to HIV-1 replication, Vpr can regulate NF-κB activity by controlling TRIM11 in HEK293 cells (Figure S4). Therefore, the physiological relevance of the regulation by Vpr of TRIM11 in terms of influencing viral reverse transcripts needs to be clarified via multi-cycle HIV-1 infectivity in non-dividing cells, in which Vpr plays an important role in viral replication.

Our study is not the only case in which different levels of Vpr can have distinct effects. For example, Wen et al. discovered that Vpr could degrade UNG2 at low expression levels but enhanced its accumulation in the cell nucleus at high expression levels [27]. That study also found that Vpr could enhance the interaction between UNG2 and the ubiquitin ligase complex CRL4^DCAF1^ [27]. However, this was not the case in our research, as suggested by the following evidence: first, TRIM11 did not directly interact with either Vpr or VprBP. Second, the MG132 treatment and VprBP knockdown experiments indicate that the ubiquitin system is indispensable for the regulation of TRIM11 by Vpr. Last, we failed to observe the sequestering of TRIM11 in the nucleus even at high concentrations of Vpr (data not shown). Other than the proteasomal degradation of UNG2, Vpr may interfere with endogenous UNG2 at the transcriptional level [42]. It has also been reported that DCAF1 is dispensable for Vpx in rescuing HIV-1 from an interferon-induced state [50]. Thus, other mechanisms might exist that serve the function of Vpr in regulating TRIM11. Although the detailed mechanism is still unclear, together with the observations that are described in our work, the concentration of Vpr may be a key to its functioning.

In summary, our study demonstrated that TRIM11 restricted the early steps of HIV-1 transduction and resulted in severely impaired reverse transcripts. We also demonstrated its role in the inhibition of HIV-1 LTR. Hence, other than acting as a negative regulator of innate immunity as reported previously, TRIM11 also acts as an antiviral factor. In addition, our results showed that TRIM11 protein levels were under the regulation of Vpr in an indirect manner that was independent of the canonical ubiquitin system. These data have implications for a better understanding of the interaction between HIV-1 and host cells and provide a basis for further work to explore the contribution of Vpr to viral replication.

## Supporting Information

Figure S1 **Effects of ectopically expressing TRIM11 on cell proliferation and cell viability.** **A**. HEK293 cell lines stably expressing RDT11, T11HA and pCDH were seeded in 12-well plates with identical concentration (∼10^5^ cells/ml). Cell numbers were counted after indicated period of time. Error bars represent the standard deviations from four independent replicates of the same experiment. **B**. HEK293 cells were transfected with 900 ng/µl control pCMV-Myc vector or Myc-TRIM11 for the indicated time period. Relative cell viability was examined by MTT assay using HEK293 cells transfected with pCMV-Myc for 24 h as control (100%).
(TIF)

Figure S2 **Effects of different HIV-1 proteins on TRIM11 protein levels.** **A**. HEK293 cells were cotransfected with different HIV-1 protein expression plasmids along with the TRIM11 expression plasmids, and cell lysates were immunoblotted with the indicated antibodies at 24 h post-transfection. **B**. HEK293 cells were cotransfected with TRIM11 expression plasmids and increasing amounts of Tat-expressing plasmids, and cell lysates were immunoblotted with the indicated antibodies at 24 h post-transfection. The numbers under each lines display the relative ratios between the Myc signals and actin signals. Representative results from three separate experiments are shown.
(TIF)

Figure S3 **Effects of Vpr on HIV-1 transduction in HEK293 cells.** **A**. HEK293 cells were inoculated with various amounts of HIV-1 Vpr^−^ and HIV-1 Vpr^+^. Luciferase assays were performed at 24 hpi. **B**. HEK293 cells stably expressing TRIM11 or a control pCDH vector were inoculated with various amounts of HIV-1 Vpr^−^ and HIV-1 Vpr^+^ viruses. Luciferase assays were performed at 24 hpi. Data are presented as fold-changes compared with TRIM11 restriction rates. Error bars represent the standard deviations from three independent replicates of the same experiment.
(TIF)

Figure S4 **Vpr bidirectionally regulates NF-κB activity via TRIM11.** **A**. HEK293 cells were cotransfected with different amounts of Vpr-expressing plasmids along with 50 ng of NF-κB firefly luciferase reporter and 50 ng of *renilla* luciferase plasmid. Luciferase assays were performed at 24 h post-transfection. Cell lysates were immunoblotted with the indicated antibodies. **B**. HEK293 cells were cotransfected with 900 ng of Vpr expressing plasmids, 50 ng of NF-κB firefly luciferase reporter and 50 ng of *renilla* luciferase plasmid along with control siRNA or TRIM11 siRNA#2 for 24 h and assayed for luciferase activity. Cell lysates were immunoblotted with the indicated antibodies. The numbers under each lines display the relative ratios between the TRIM11 signals and actin signals. Error bars represent the standard deviations from three independent replicates of the same experiment. *P<0.05.
(TIF)
