# Supplementary figures and images for: The Human Antiviral Factor TRIM11 Is under the Regulation of HIV-1 Vpr
Source: PLoS One. 2014 Aug 8;9(8):e104269. doi: 10.1371/journal.pone.0104269 (PMC4126725; doi:10.1371/journal.pone.0104269)

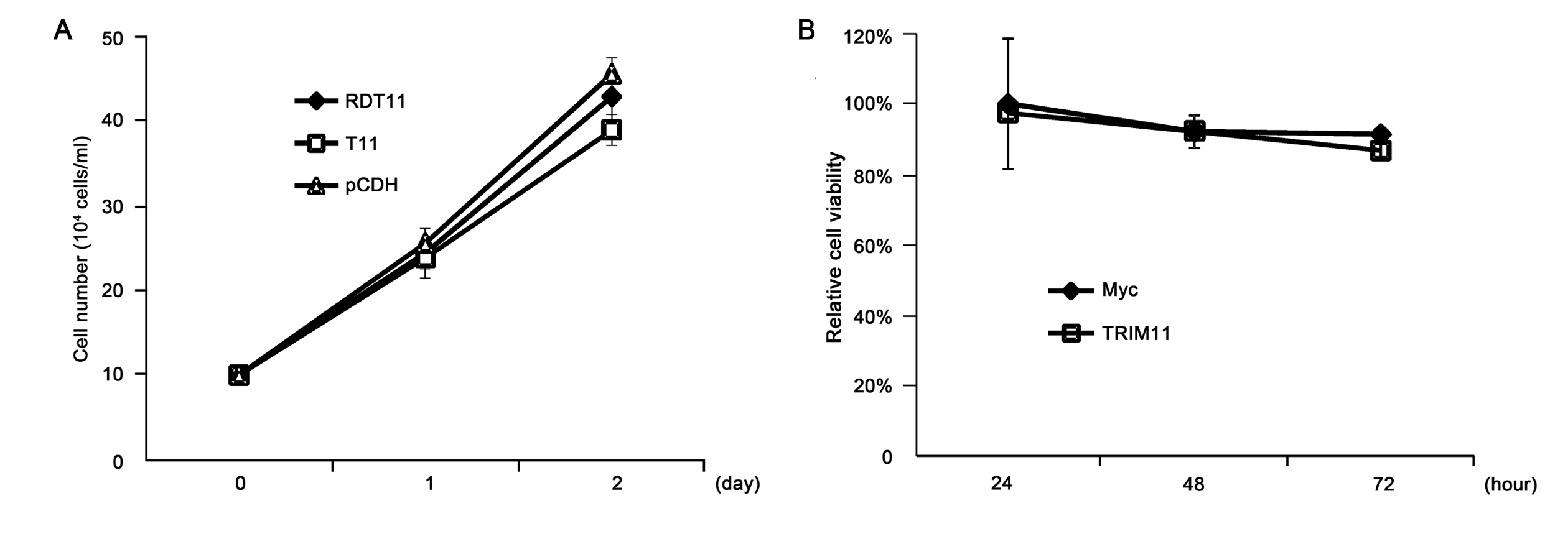

Supplement: Figure S1 — Effects of ectopically expressing TRIM11 on cell proliferation and cell viability. A. HEK293 cell lines stably expressing RDT11, T11HA and pCDH were seeded in 12-well plates with identical concentration (∼105 cells/ml). Cell numbers were counted after indicated period of time. Error bars represent the standard deviations from four independent replicates of the same experiment. B. HEK293 cells were transfected with 900 ng/µl control pCMV-Myc vector or Myc-TRIM11 for the indicated time period. Relative cell viability was examined by MTT assay using HEK293 cells transfected with pCMV-Myc for 24 h as control (100%). (TIF) [file pone.0104269.s001.tif]

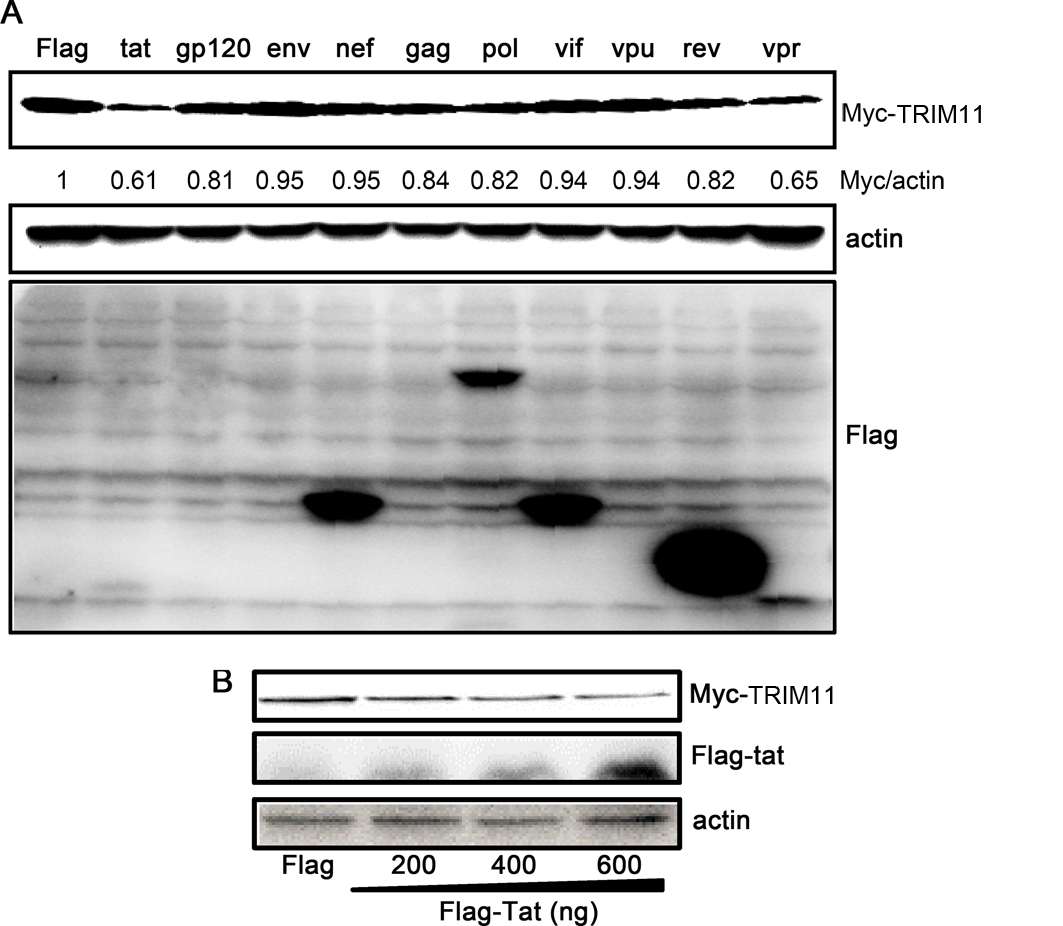

Supplement: Figure S2 — Effects of different HIV-1 proteins on TRIM11 protein levels. A. HEK293 cells were cotransfected with different HIV-1 protein expression plasmids along with the TRIM11 expression plasmids, and cell lysates were immunoblotted with the indicated antibodies at 24 h post-transfection. B. HEK293 cells were cotransfected with TRIM11 expression plasmids and increasing amounts of Tat-expressing plasmids, and cell lysates were immunoblotted with the indicated antibodies at 24 h post-transfection. The numbers under each lines display the relative ratios between the Myc signals and actin signals. Representative results from three separate experiments are shown. (TIF) [file pone.0104269.s002.tif]

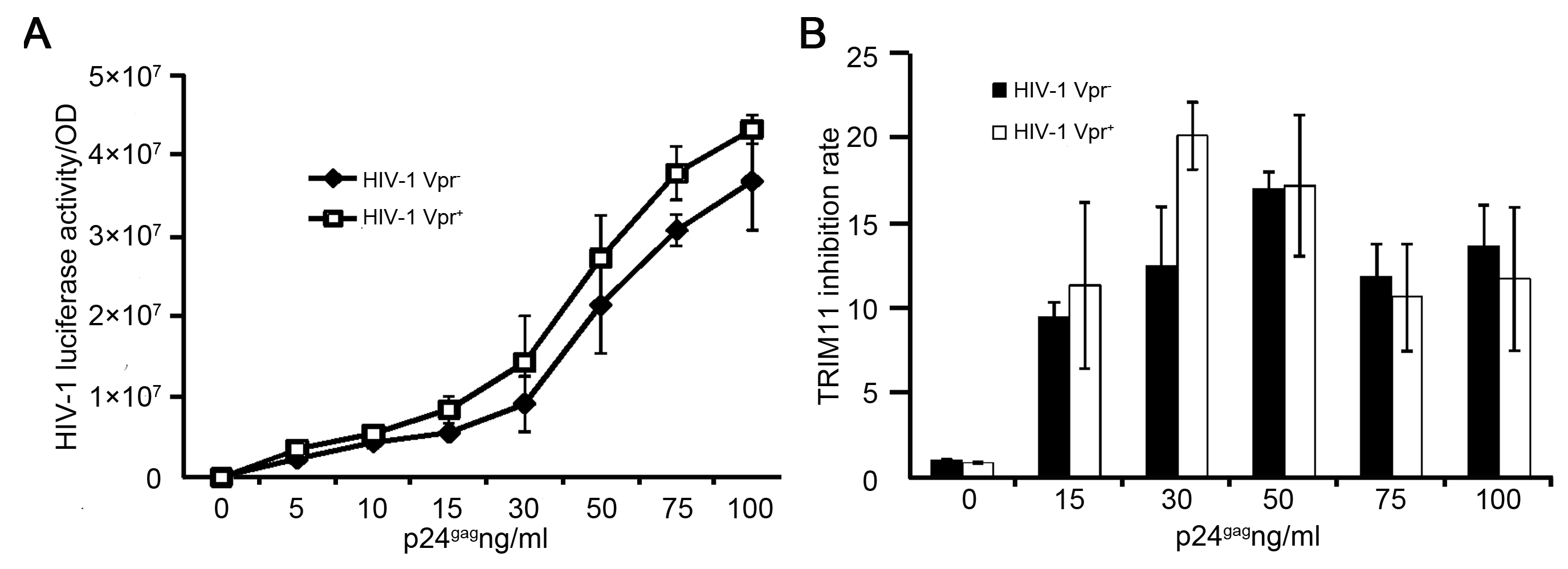

Supplement: Figure S3 — Effects of Vpr on HIV-1 transduction in HEK293 cells. A. HEK293 cells were inoculated with various amounts of HIV-1 Vpr− and HIV-1 Vpr+. Luciferase assays were performed at 24 hpi. B. HEK293 cells stably expressing TRIM11 or a control pCDH vector were inoculated with various amounts of HIV-1 Vpr− and HIV-1 Vpr+ viruses. Luciferase assays were performed at 24 hpi. Data are presented as fold-changes compared with TRIM11 restriction rates. Error bars represent the standard deviations from three independent replicates of the same experiment. (TIF) [file pone.0104269.s003.tif]

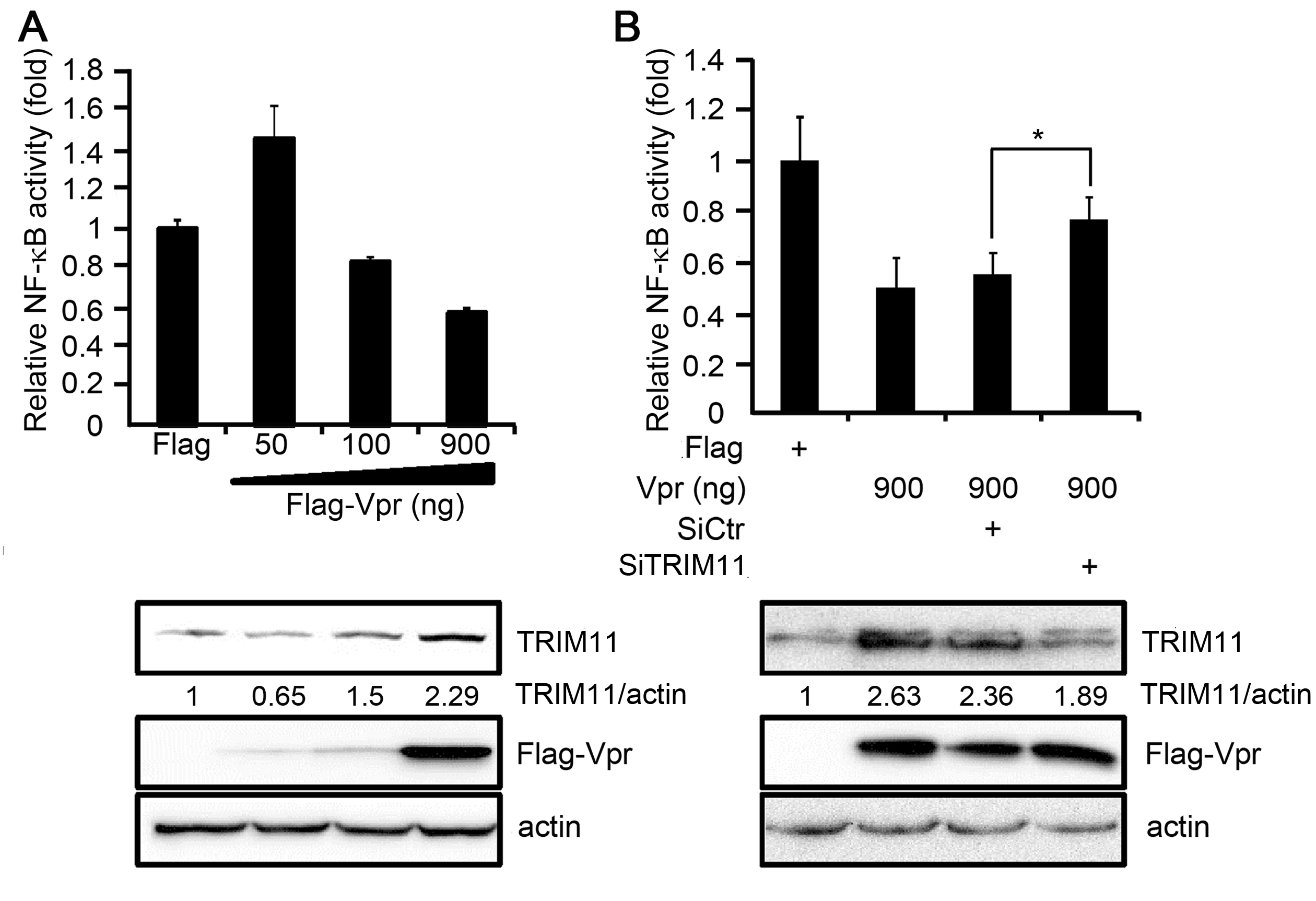

Supplement: Figure S4 — Vpr bidirectionally regulates NF-κB activity via TRIM11. A. HEK293 cells were cotransfected with different amounts of Vpr-expressing plasmids along with 50 ng of NF-κB firefly luciferase reporter and 50 ng of renilla luciferase plasmid. Luciferase assays were performed at 24 h post-transfection. Cell lysates were immunoblotted with the indicated antibodies. B. HEK293 cells were cotransfected with 900 ng of Vpr expressing plasmids, 50 ng of NF-κB firefly luciferase reporter and 50 ng of renilla luciferase plasmid along with control siRNA or TRIM11 siRNA#2 for 24 h and assayed for luciferase activity. Cell lysates were immunoblotted with the indicated antibodies. The numbers under each lines display the relative ratios between the TRIM11 signals and actin signals. Error bars represent the standard deviations from three independent replicates of the same experiment. *P<0.05. (TIF) [file pone.0104269.s004.tif]
